# Colonization process determines species diversity via competitive quasi‐exclusion

**DOI:** 10.1002/ece3.7342

**Published:** 2021-03-16

**Authors:** Atsushi Yamauchi, Koichi Ito, Shota Shibasaki

**Affiliations:** ^1^ Center for Ecological Research Kyoto University Otsu Japan; ^2^ Department of Zoology University of British Columbia Vancouver BC Canada; ^3^ Department of Fundamental Microbiology University of Lausanne Lausanne Switzerland

**Keywords:** coexistence, community, rank abundance diagram, statistical test, theory

## Abstract

A colonization model provides a useful basis to investigate a role of interspecific competition in species diversity. The model formulates colonization processes of propagules competing for spatially distinct habitats, which is known to result in stable coexistence of multiple species under various trade‐off, for example, competition–colonization and fecundity–mortality trade‐offs. Based on this model, we propose a new theory to explain patterns of species abundance, assuming a trade‐off between competitive ability and fecundity among species. This model makes testable predictions about species positions in the rank abundance diagram under a discrete species competitiveness. The predictions were tested by three data of animal communities, which supported our model, suggesting the importance of interspecific competition in community structure. Our approach provides a new insight into understanding a mechanism of species diversity.

## Graphical abstract

In the present study, we show that colonization model can explain species diversity in ecological communities, which represents typical trends observed in the rank abundance diagrams (RADs) and provides testable predictions about species positions in the RAD. We test those with three literature data, which indicates that our model may be supported by empirical data. This is a significant study that suggests a mechanism of species diversity based on statistical tests with labeling on RAD.

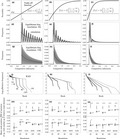

## 1 INTRODUCTION

Species abundance distribution (SAD) is an important ecological concept because it characterizes the properties of species diversity within communities. One type of SAD, a histogram of species abundance, was first introduced by Preston (1948). Thereafter, it was widely used to describe patterns in community structures (May, 1975; Pielou, 1975), which show a universal trend of a hyperbolic convex curve with few abundant species and many rare species. Another type of SAD plot proposed by Motomura (1932) is the rank abundance diagram (RAD), in which log abundance is plotted against the abundance rank of species. It can illustrate contrasting patterns of species richness and highlight unevenness among assemblages. RAD shape is often used to infer which model best describes species abundance data (Magurran, 2004; Whittaker, 1965; Wilson, 1991).

Many hypotheses have been proposed to explain the observed patterns of SADs. McGill et al. listed 27 different models, which were classified into five categories: purely statistical, branching processes, population dynamics, niche partitioning, and spatial distribution (Matthews & Whittaker, 2014; McGill et al., 2007). SADs have also been studied using population dynamics models with respect to random communities (Tokita, 2015). Despite numerous attempts, it remains unclear which mechanisms are key determinants in species composition, and whether fitness is a significant factor. McGill et al. (2007) emphasized the need for distinct predictions that can be tested, because most theoretical studies explain the shape of abundance graphs without additional predictions, so‐called goodness‐of‐fit tests (Magurran, 2004; Sugihara et al., 2003). One possible direction to help overcome this problem is an integrative comparison of model predictions across many different patterns, for example, various SADs, species–area relationship, and beta‐diversity (Hubbell, 2001; Levin, 1992; May et al., 2015; McGill et al., 2007). Matthews and Whittaker (2014) also recommend the use of both goodness‐of‐fit tests and model‐comparison analyses for evaluation of SAD models. Another direction is an extraction of more detailed information from SAD by referring species identities, that is, ‘labeled’ SAD (McGill et al., 2007). This approach is not irreconcilable with the integrative comparison of patterns, simultaneous developments of which could rather contribute to progress understanding properties of community structures.

The ‘labeling’ approach has been adopted to study empirically observed SADs, focusing on various traits. Species abundance was sometime investigated with information of species size (see a review by White et al. (2007)). For example, Russo et al. (2003) studied size–abundance relationship in bird community, that is, labeling of size, which suggested that spatial distribution of resources and interference competition within guilds may explain patterns of the observed relationship. Murray et al. (1999) labeled species identities and attributes on RAD and investigated properties of flowering plant species that are rare throughout their geographical range. Although they cannot find clear tendency, Murray and Westoby (2000) developed this study, showing significant differences between low‐ and high‐abundance plant species in seed production and population structure. Sugihara et al. (2003) labeled species niche in empirically reported communities, which indicated that shapes of SAD tend to correlate with shapes of dendrogram of niche similarity, being consistent with a prediction of broken stick model. Shipley et al. (2006) investigated relative abundance of species by focusing on 8 functional traits of 30 plant species, which showed that those traits influenced species abundance via species sorting in the communities. These studies with ‘labeling’ indicated an importance of species identity in study of biodiversity. In particular, this approach could provide much information to understand community structures in the presence of species interactions, for example, interspecific competition.

Interspecific competition spreads in nature (Connell, 1983; Schoener, 1983) and might be a significant factor to community structure in some systems (e.g., plant communities). In the 1960s and early 1970s, competitive interactions were regarded as the preeminent process in determining community structure (Cody & Diamond, 1975; MacArthur, 1972). Nevertheless, in the late 1970s and 1980s, there emerged questions about the lack of tests to reject null hypotheses that the random assemblage of species without interspecific competition could represent consistent patterns to observed communities, as well as about the validity of assumptions in the equilibrium theory of competition (Connor & Simberloff, 1979; Strong et al., 1984). The role of interspecific competition in species diversity may have been disregarded since then and even strongly rejected (Rohde, 2005).

Despite such negative views, some theoretical researches indicated that the competition can contribute species diversity under specific conditions. By analyzing Lotka–Volterra competition model in metacommunities, O'Sullivan et al. (2019) found that competitive interaction can promote biodiversity in a metacommunity and that their model can replicate community patterns similar to empirical observations, that is, uneven SAD, skewed range size distribution, and nonsignificant correlation in species' spatial distributions. These results may, however, depend on the high immigration rates (with a low biomass) of new species from outside the communities in their model.

Meanwhile, colonization model was proposed to investigate species coexistence under competitive interactions (Hastings, 1980; Levins & Culver, 1971), by formulating the colonization processes of propagules competing for spatially distinct habitats. It was assumed that when two species encounter in a site, the competitively superior species always immediately defeats the competitively inferior species in a scheme called ‘displacement competition’ (Yu & Wilson, 2001) or ‘dominance competition’ (Calcagno et al., 2006). The analyses revealed that colonization processes can promote the coexistence with various trade‐offs between species properties (e.g., competition–colonization and fecundity–mortality trade‐offs). Indeed, colonization of woody plants affects plant community composition (Collins et al., 2009; Cook et al., 2005; Schweiger et al., 2000), suggesting relationships between colonization process and species diversity. Colonization processes can be a mechanism of species coexistence, accompanied by a certain shape of RAD (Kinzig et al., 1999; Lehman, 2000; Tilman, 1994), although the relationship between colonization and SAD has not been clearly studied. The colonization model was not included in the list of SAD studies (e.g., McGill et al., 2007); it may be considered a less important hypothesis due to an insufficient mention to RAD shape.

It is worthwhile to analyze a role of interspecific competition in species diversity by focusing on colonization process in relation to SAD in detail. The combination of colonization model and labeling approach could clarify a relationship between interspecific competition and community structure. To reveal the contribution of competition to species diversity, we extended a colonization model with a competition–fecundity trade‐off, combining with the labeling approach.

## 2 MODEL

We constructed a mathematical model based on formulas in previous studies (Hastings, 1980; Kinzig et al., 1999; Levins & Culver, 1971; Tilman, 1994). A community is assumed to involve *n* species, although some may go extinct. The species are indexed by *i* = 1, 2,…*n*, which coincides with the order of their competitive ability, that is, a small *i* represents the competitively superior species, considering competitiveness as a discrete property. The habitat consists of many sites that are suitable for those species, and each site is either empty or colonized by a single species. The colony continuously reproduces, and offspring disperse to colonize other sites. Since competitiveness may be costly, it reduces colony reproduction; the fecundity of the *i*‐th species, *f_i_*, increases with index *i* (decreasing competitiveness), that is, competition–fecundity trade‐off, as suggested by some empirical studies (Ghalambor & Martin, 2001; Rees et al., 2001).

The dispersing offspring will encounter sites with an encounter rate, *q*. When they encounter an unoccupied site, a new colony will successfully establish there. When they encounter an occupied site, they will compete with the site owner and the competitively superior species will immediately occupy the site following the ‘dominance rule’. A site with an established colony will become destructed due to environmental disturbances or attacks from higher‐trophic level of species at rate *m*. Under discrete competitiveness, the continuous‐time dynamics of site frequency with *i*‐th species, *p_i_*, can be expressed by the formula:

(1) $$\frac{dp_{i}}{\mathrm{dt}}=qf_{i}p_{i}\left(1‐\sum_{j=1}^{i}p_{j}\right)‐q\left(\sum_{j=1}^{i‐1}f_{j}p_{j}\right)p_{i}‐mp_{i},$$

as the previous studies (Kinzig et al., 1999; Tilman, 1994). To extend the model for a case with continuous competitiveness, we consider that the *i*‐th species is characterized by competitive inferiority, *x_i_*, which is a continuous value that is larger for less competitive species, that is, *x*_1_ < *x*_2_ < … < *x_n_*. Based on this definition, fecundity *f_i_* can be a monotonically increasing function of competitive inferiority *x_i_* as *f_i_* = *f*(*x_i_*), which is a competition–fecundity trade‐off function. In the dynamic equation, the first term represents an increment of a colony of the *i*‐th strain by colonizing sites that are empty or occupied by inferior species, whereas the second term is a decrement due to occupation by a superior species. The last term indicates colony extinction from disturbances or attacks of higher‐trophic species.

From Appendix A, the equilibrium solution of this system is given by:

(2) $$p_{i}=\left\{\begin{array}{cc} 1‐\sum_{j=1}^{i‐1}p_{j}‐\frac{1}{f_{i}}\left(\sum_{j=1}^{i‐1}f_{j}p_{j}+\frac{m}{q}\right) & \text{if}\;\text{it}\;\text{is}\;\text{positive}\\ 0 & \text{otherwise} \end{array}\right),$$

as the previous studies (Kinzig et al., 1999; Tilman, 1994). This shows that *p_i_* can be determined from *f_i_* and information on species with smaller indexes *j* < *i*. This implies that the frequency of all species can be determined numerically by a forward recursive procedure from *i* = 1 to *n*. Importantly, when the fecundity *f_i_* increases with *i*, the first species appears at the first point that satisfies the condition *f_i_* > *m*/*q* in the forward procedure.

By taking an infinite number of species as *n*→∞, the discrete competitiveness may be regarded as continuous under continuous trade‐off function *f_i_* = *f*(*x_i_*). An equilibrium frequency distribution of the continuous competitiveness, *p*(*x*), can be derived analytically as a density function. Kinzig et al. (1999) derived the solution for a linear trade‐off. By extending their approach, a general solution can be obtained for any competition–fecundity trade‐offs (see Appendix B) as:

(3) $$p\left(x\right)=\frac{1}{2}\frac{f^{\prime }\left(x\right)}{{\left\{f(x)\right\}}^{3/2}}\sqrt{\frac{m}{q}}\;\text{for}\;x_{c}\leq x\leq \hat{x},$$

where *x_c_* and $\hat{x}$ are the solutions of *f*(*x_c_*) = *m*/*q* and a maximum competitive inferiority in the considered system, respectively. Since Eq. (3) is a density function of frequency, the substantial frequency is derived by integrating it within a given interval of competitive inferiority, which is expressed by:

(4) $$p\left(x;x+\Delta x\right)=\left(\frac{1}{\sqrt{f(x)}}‐\frac{1}{\sqrt{f(x+\Delta x)}}\right)\sqrt{\frac{m}{q}},$$

with an interval from *x* to *x* + *∆x*. This can be a baseline of species distribution in the following analysis.

On the other hand, since a real community does not include an infinite number of species in general, we should consider a finite number of species with discrete values of competitiveness. With discrete competitiveness, serrated patterns occur in equilibrium frequency distributions. This trend occurs because the frequency of species with the lowest competitive inferiority beyond *x_c_* is unlikely to correspond with analytical solutions in numerical analyses. When the species achieves a higher (or lower) frequency than the analytical solution, the next species occurs in a lower (or higher) frequency due to intense (or temperate) competitive pressure. This effect propagates recursively from less inferior species to more inferior species, causing the serrated frequency distributions.

## 3 RESULTS

### 3.1 Model solutions

**FIGURE 1:**
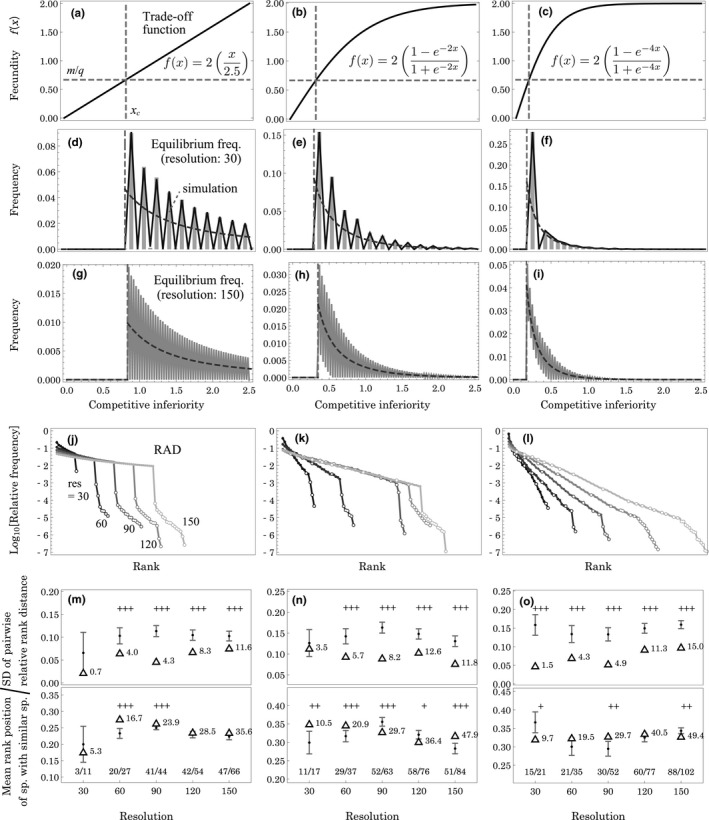
Species composition properties under various competition–fecundity trade‐offs (a–c). Solutions of equilibrium frequency with resolutions of (d–f) *n* = 30 and (g–i) *n* = 150 within 0 ≤ *x* ≤ $\hat{x}$ = 2.5, respectively. Lines represent the numerical solutions in discrete competitiveness, and broken curves are the analytical solutions to continuous competitiveness. Panels (d–f) also plot the results of simulations. Panels (j–l) are rank abundance diagrams for discrete competitiveness under various resolutions with ignoring species with frequencies lower than 10^−7^, where solid and open dots represent peak and nonpeak species, respectively. The peak species is a species that achieves higher frequency than both its neighboring species on the competitiveness axis. Panels (m–o) plot two indexes, the standard deviations (SDs) of pairwise rank distances between species adjoined in competitiveness (upper panel) and the average rank positions of species that survive with similar species (lower panel), which are derived from the relative rank positions to resolution. The triangles indicate the index values of modeled cases, and labels indicate values based on absolute rank positions. Dot plots with error bars represent the means and SDs of the corresponding indexes in 5,000 trials of randomization. + denotes significance (see text). The fractions at the bottom represent the number of species surviving together with neighboring species in competitiveness (numerator) and the numbers of surviving species (denominator)

Figure 1 illustrates examples of solutions to the equilibrium frequency distributions of species for both discrete and continuous competitiveness. Figure 1a–c plots the competition–fecundity trade‐off functions, which are assumed to be linear and saturating functions. Saturation of the trade‐off occurs because maximum fecundity is limited by environmental factors, which cannot be exceeded even with a significant reduction in competitive ability (effects of functional forms of trade‐off are also discussed in Discussion section below). Figure 1d–i indicates the solutions of equilibrium frequency for *n* = 30 and 150, respectively, when the trade‐off function is given, as in Figure 1a–c. Here, species are assigned competitive inferiority *x_i_* with even intervals within 0 ≤ *x* ≤ $\hat{x}$ = 2.5. In those figure panels, broken gray curves indicate the analytical solutions in continuous competitiveness. Solid lines represent numerical solutions in discrete competitiveness. Additionally, Figure 1d–f plots the results of a simulation by gray bar charts, which fit the numerical solutions.

The analytical solutions in continuous competitiveness represent continuously smooth distributions, suggesting that an infinite number of species can occur within a given range of competitive ability, which is consistent with a previous study for a linear trade‐off (Kinzig et al., 1999). Such solutions may be unrealistic because a natural community does not include an infinite number of species. It could appear as a finite number of discrete trait if major reductions of fecundity are necessary to increase competitiveness, the magnitude of which may be environmentally determined. In this sense, the number of potential species *n* can be regarded as a resolution within the given range, which is determined by the cost of competitive ability. Namely, a lower resolution implies a larger cost.

Figure 1d–i shows that the equilibrium frequency is not gradual with discrete competitiveness, unlike with continuous competitiveness. Rather, the serrated distributions are due to low occupancy or extinctions of species, a trend that remains even with large *n*, that is, a fine resolution (see Figure 1g–i). Such a characteristic pattern is caused by intense competition between neighboring species. It should be noted that a similar pattern was also partly reported in simulations of an explicitly spatial version of colonization model with an exponential trade‐off (Lehman, 2000). The mechanism forming serrated pattern is similar to competitive exclusion, although it is likely to result in coexistence of neighboring species rather than the extinction of either species. Therefore, we refer it hereafter by ‘competitive quasi‐exclusion’.

The frequency distribution of a species can be translated to a rank–abundance diagram, RAD (Kinzig et al., 1999). Figure 1j–l illustrates RADs for discrete solutions with varying resolutions of competitiveness under given competition–fecundity trade‐offs, in which frequencies are normalized by excluding empty sites and species with frequencies lower than 10^−7^. In those panels, there are two types of species, peak and non–peak species represented by solid and open dots, respectively. A peak species achieves a higher frequency than species on either side of the competitiveness axis. Accordingly, the RADs often consist of two phases, where peak and non‐peak species form clusters at higher and lower ranks, respectively (Figure 1j). A significant gap can appear between two phases (Figure 1j,k), depending on the functional form of trade‐off. The change in RAD shape results from two properties of the trade‐off curves: a steeper trade‐off results in a steeper decrement of RAD, and a strong saturation of trade‐off reduces the gap. Consequently, various forms of RAD curves depend on the competition–fecundity trade‐off. A decrement of resolution results in a short and steep RAD, which suggests that a different RAD shape is partly caused by variation in the trade‐off intensity. In either case, the RADs are similar to empirically observed RAD trends, that is, a reduction of logarithmic abundance is approximately linear at a high ranks, but is accelerated at low ranks (Wilson, 1991).

**FIGURE 2:**
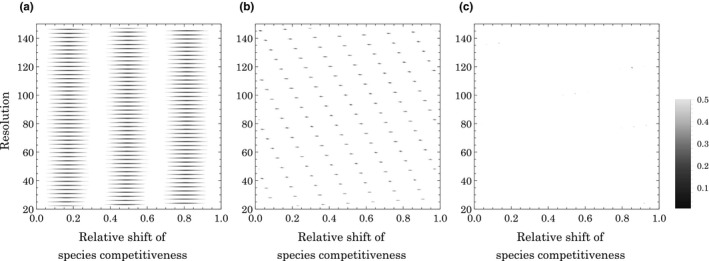
Variability of frequency distributions in discrete competitiveness under various combinations of resolution and relative shifts of species competitiveness. The species' positions are determined by [original position] + [relative shift] × [absolute interval between species ($\hat{x}/n$)], where the relative shifts changes from 0 to 1 with 0.001 interval. Panels (a–c) are the results of the trade‐off functions illustrated in Figure 1a–c, respectively. The variability of the frequency distribution is denoted by an average ratio of the difference between the analytical and numerical solutions to the analytical solution in species frequency. This indicates that variability tends to be greater than 20% under the broad conditions

Species distribution can notably change with shifting species competitiveness, even while maintaining their intervals. Note that the horizontal shift in species position corresponds with a shift in trade‐off function in the opposite direction and is therefore equivalent to a modification of the *f*(*x*) curve. An example of shifting the species competitiveness positions is illustrated in Figures S1, which shows that the shift influences the variability of frequency distributions in discrete competitiveness. In Figures S1d,g with a linear trade‐off, the frequency distributions with discrete competitiveness are likely to coincide with those with continuous competitiveness, resulting in simply convex RADs. To examine the effects of such shift, we checked the variability of distributions with changing resolutions (*n*) and a relative shift in species position under various trade‐off functions because a serrated species frequency is necessary for the characteristic shape of the RAD. Figure 2 indicates that the fluctuation of species frequency generally occurs with discrete competitiveness, but can be small especially with a linear trade‐off. A previous study showed an example where all species coexist continuously under a linear trade‐off function (Kinzig et al., 1999), although our result suggests that may not be a general trend. Despite such exceptions, the frequency distribution occurs in a serrated pattern especially under nonlinear trade‐off functions with discrete competitiveness.

**FIGURE 3:**
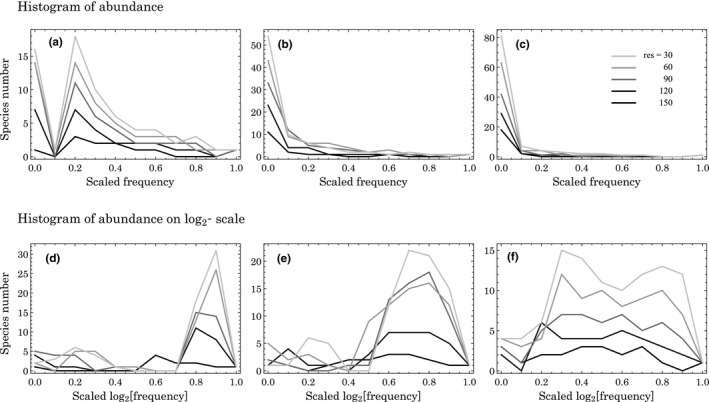
Two types of SADs that are translated from Figure 1j–k. Panels (a–c) are simple histograms of species frequencies, whereas panels (d–f) are histograms on log_2_‐scale. Ranges of species frequencies are normalized between 0 (the minimum frequency) and 1 (the maximum frequency), in which species frequencies are categorized into 11 classes with a 0.1 interval

In Figure 1j–k, we illustrate RAD of analyzed communities. In order to confirm properties of community structure in other SADs, we translate the RADs into two types of SADs, that is, simple histograms of species frequencies (Figure 3a–c) and histograms of species frequencies on log_2_‐scale (Figure 3d–f). According to the figure, when the trade‐off is a saturating function, the simple histograms tend to represent hollow curves with long tail (Figure 3b,c), whereas the histograms on log_2_‐scale show a spread of species distributions toward low abundance region (Figure 3e,f). These patterns may be consistent with trends that are sometimes observed in natural communities (Hubbell, 2001; McGill et al., 2007).

### 3.2 Model predictions via labeling species identity

The present analysis may represent trends in RAD shapes that have been empirically observed, although it is a prediction based on similarity to empirical data in graphical shape, which has been argued as a central problem of theoretical studies in species abundance, that is, a lack of testable prediction (McGill et al., 2007; Sugihara et al., 2003). However, the present model is unique because it can make testable predictions on characteristics other than RAD shape, by focusing on ecological similarities of species that survives in the community. In this case, ecological similarity is denoted by a proximity of competitiveness and fecundity on the trade‐off function. Here, we label species on RAD with respect to the similarity.

In the colonization process with a competition–fecundity trade‐off, the frequency distribution of species shows a serrated pattern on the competitiveness axis. This can result in characteristic trends of species distributions in RADs, where peak species persist and nonpeak species either go extinct or survive due to competitive quasi‐exclusion. It should be noted that frequencies of peak species tend to be similar with each other and that those of surviving nonpeak species are also similar with one another. Rearranging the surviving species can result in distributions of peak and nonpeak species at high and low ranks on the RAD, respectively. Since the species frequencies gradually vary within groups, RAD tends to maintain the orders within peak and nonpeak species, respectively. Thus, if pairs of species adjoining in the competitive axis survive simultaneously, those pairs (i.e., peak and nonpeak species) tend to represent similar pairwise distances in RAD, which results in a small variation of the pairwise distances. Therefore, variation in rank distances between surviving species that adjoin on competitive axis could be an index of effectivity of interspecific competition over the community.

Furthermore, the serrated pattern also influences the position of species on RAD that survive together with neighboring species on a competitive axis, which can result in a bias of rank distribution of those species through three processes. The distribution can be affected by the extinction of nonpeak species concentrated at a limited range of competitiveness. First, intense competition could cause the extinction of nonpeak species within a range of relatively high competitiveness (e.g., Figure 1h). In this case, both peak and nonpeak species are likely to survive with relatively low frequencies at regions of low competitiveness, which skews the distribution of species with surviving neighbors toward the low ranks. Second, when a species frequency rapidly decreases with an increment of competitive inferiority, extinction tends to occur in nonpeak species with low competitiveness (e.g., Figure 1i). This suggests that peak species with low competitiveness lose neighboring nonpeak species. Such peak species are assigned a low rank due to low competitiveness; therefore, the number of species with surviving neighbors declines at the low‐rank range and biasing the distribution toward the high rank range on RAD. Finally, the bias can occur from a difference in the fate of peak and nonpeak species. Neighborhoods of peak species are nonpeak species that might be extinct, whereas those of nonpeak species are peak species that generally can survive. Since the surviving nonpeak species tend to represent low ranks due to their low frequencies, nonpeak species with surviving neighbors could distribute at a low‐rank range on the RAD. This is the basal biasing effect of the serrated pattern on the RAD.

By randomizing species identities in RAD, we examined the probabilities that the observed *SD* of rank distances between adjoining species is significantly smaller than that of randomization and that the average rank position of species with neighboring species is significantly different from that of randomization. The latter probability is calculated as a conditional probability because the difference is evaluated regardless of the magnitude relation between two comparable values. If the average rank position of the analysis is less or greater than the mean within‐trial randomized averages, the significance is examined by extracting trials with a within‐trial average that is less or greater than the mean within‐trial averages, respectively (i.e., an analogy to two‐sided test in symmetric probability distribution). In Figure 1m–o, triangles show indexes for those in example cases. The values are based on the relative rank position to resolution, although the labels show absolute rank positions. Meanwhile, plots with an error bar represent mean values and SDs of the corresponding indexes in 5,000 randomized trials that shuffled species identities in RAD. In Figure 1m–o, +++, ++, and + show that the observed *SD* of rank distance occurs within a range of 5%, 10%, and 15% of smallest randomized values in the upper panels and that the observed rank position occurs within a range of 5%, 10%, and 15% of the smallest or largest randomized values in the lower panels. According to the results, the *SD* of pairwise rank distances tends to be significantly smaller than randomization, whereas the average rank position is not always, but sometimes, significantly different from those of randomization.

It should be remarked that we consider regular intervals of competitive ability in Figure 1. This may appear to be an unrealistic assumption, but regular intervals can be justified because competitiveness represents the relative superiority during competition. Since the result of competition is determined by the order of competitive ability, regardless of the absolute values, species positions can be relocated on the competitiveness axis while maintaining their rank orders. This means that the interval of discrete competitiveness can be regularized by adequately choosing the trade‐off function. Even in such cases, some fluctuations might be possible in trade‐off function. We supplementarily examine the effects of fluctuations in trade‐off by introducing operational randomness to specific competitiveness, *x_i_*.

To examine the robustness of our result, we checked the effects of randomness on equilibria and RADs, in which species competitiveness randomly fluctuates from its expected position within a range of ±interval/2, and fluctuates completely at random within 0 ≤ *x* ≤ $\hat{x}$ (=2.5) (Figures S2 and S3, respectively). The results show that a small perturbation makes species abundance more even, although the overall trend of RADs does not vary (compare Figure 1j–l to Figure S2j–l). Meanwhile, a large perturbation produces unclear trends due to significant disturbances in the trade‐off function (see Figure S3j–l), where the trade‐off relationship may become indefinite. Tendencies in species position on RAD are also tends to be maintained to some degree in cases with random positions of competitiveness (Figures S2 and S3). It should be noted that Figures S2 and S3 are results of limited examples of random competitiveness; therefore, those are only references about indices in randomly constructed communities.

### 3.3 Tests on empirical data

**FIGURE 4:**
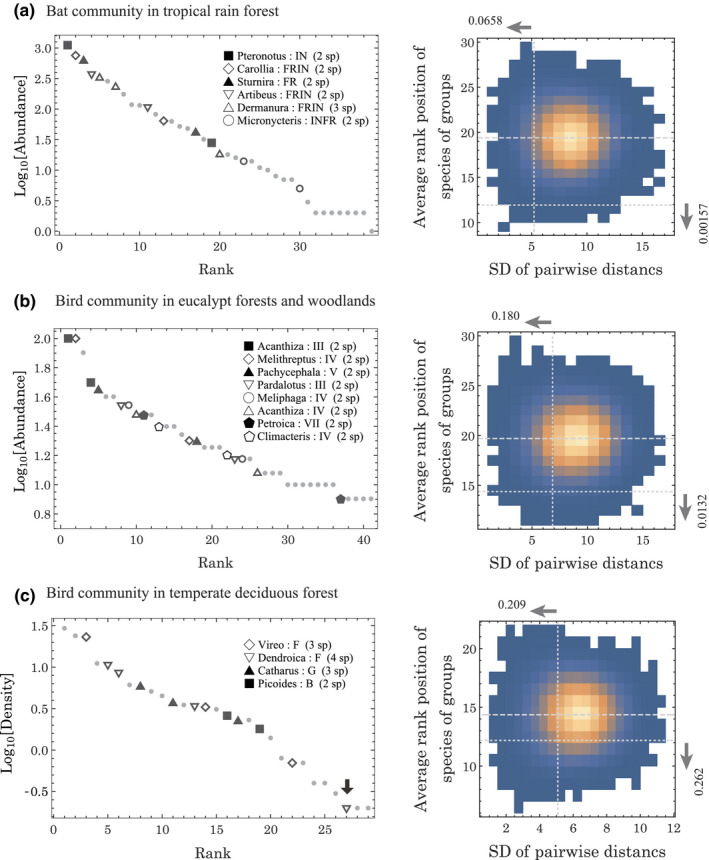
Rank abundance diagrams of three communities that were observed empirically, and the distributions of the average and standard deviation of pairwise distances between species within a group after randomization of species rank. (a) A bat community with 39 species in the tropical region of Los Tuxtlas, Mexico (Estrada & Coates‐Estrada, 2001), (b) a bird community with 41 species in the eucalypt forests of southeastern Australia (Holmes & Recher, 1986), and (c) a bird community with 29 species in a temperate forest of New Hampshire, United States (Holmes et al., 1986). In the left panels, each mark (except for the small light gray dot) represents a species belonging to a group of certain genera and foraging guilds, as described in the legend. In (a), the foraging guilds are categorized as IN: insectivore, FRIN: frugivore complementing diet with insects, FR: frugivore, and INFR: insectivore complementing diet with fruit. Five out six groups include two species, whereas one group (*Dermanura*: FRIN) involves three species. In (b), the Roman numerals indicate the foraging guilds, which are categorized by foraging method, substrate, and plant species (see Holmes & Recher, 1986), involving eight groups with two species. In (c), the foraging guilds are denoted by locations where the majority of foods are obtained: F: foliage of trees, shrubs, and herbs, G: ground and litter, and B: bark of tree boles and branches. Two out of four groups include three species, whereas the remaining two groups involve two and four species. In (c), a species indicated by the black arrow was excluded from analyses but included in an additional analysis. In those panels, gray dots indicate species without taxonomically or ecologically similar species. The right panels illustrate histograms of combination of two indexes of randomization trials with 100,000 iterations, excluding the species with the black arrow in (c). In those panels, brighter colors indicate a higher frequency, whereas white regions represent absence. The vertical and horizontal dotted lines show index values of the original data. Horizontal dashed lines indicate the mean rank position among all randomization trials, which is a baseline to calculate the conditional probabilities for a difference of average rank position between the original and randomized data. The numbers on the top and right sides of each panel indicate the probability that the randomized data satisfy a relevant condition (see text)

We examined predictions of our model with three empirical data: bat diversity in the tropical region of Los Tuxtlas, Mexico (Estrada & Coates‐Estrada, 2001), bird diversity in eucalyptus forests of southeastern Australia (Holmes & Recher, 1986), and bird diversity in temperate forests of New Hampshire, United States (Holmes et al., 1986). In birds species, the trade‐off between fecundity and survivorship is driven by predation risk (Ghalambor & Martin, 2001), supporting the assumption of our model. The above literatures include abundance data of multiple species and their foraging guilds. According to the data, species are grouped by genus and foraging guild, which are expected to possess similar ecological properties (see right panels, Figure 4). Consequently, the Mexican bat community involves five groups of two species and one group of three species; the Australian bird community includes eight doublets, those in the United States include one doublet, two groups of triplets, and one group of four species. Using different markers for each group, the RADs of those communities are plotted in Figure 4. In the dataset of the American bird community, the quartet involves a species with the minimum abundance rank (black arrow in Figure 4c). According to the literature, records of this species, the yellow‐rumped warbler (*Setophaga coronata*), are extremely rare. In a 16‐year census, this species was directly observed by a single individual in 3 years and indirectly detected by trace evidence in a single year, whereas other species with similar abundance were recorded by trace evidence in many years (Holmes et al., 1986). The trace evidences were not used to estimate abundance, although the rarity of trace records brings into question whether the study area was the native habitat of this species. Thus, we analyzed the data while both including and excluding the yellow‐rumped warbler.

To test our predictions, we randomized the identities of those species in RAD 100,000 times. We derived the SDs of all rank distances between pairwise species in the same group and averages of their rank positions. The data include multiple species with the same rank and abundance (see Supplementary Datasets), properties of which are preserved while measuring rank distances and randomizing species identities. Based on the randomized dataset, we calculated the product of two probabilities: if the distance *SD* is significantly smaller than at random, and if the average rank is significantly different from random (see right panels of Figure 4). Consequently, the joint probabilities of the two criteria are 0.0001, 0.0024, and 0.0549 in three data in Figure 4a–c, respectively. When including the yellow‐rumped warbler in Figure 4c, the observed *SD* of rank distance becomes greater than average *SD* of randomization with a statistically insignificant joint probability of 0.311.

Some biases could exist in the *SD* of pairwise distance within groups including more than two species. The colonization model predicts that pairwise distances of three adjoining species involve two long and one short length because the first and third species are likely to represent similar frequencies. The measurement of all pairwise distances in those groups could overestimate the *SD* compared to measurement of distances between exactly neighboring pairs. Even if overestimating, the prediction is significant in the observed dataset (marginally significant in the American bird data without yellow‐rumped warbler). Without bias, the prediction is expected to be more significant. Consequently, our analyses suggest that the trend of species abundance in observed data is unlikely to be explained by randomness, which may be inconsistent with the neutrality of species (Hubbell, 2001).

## 4 DISCUSSION

The present paper suggests that competitive interaction among species can be an important factor in determining the structure of ecological communities. The role of competition in diversity may have been overlooked and even strongly rejected (Rohde, 2005). Interspecific competition essentially results in a negative effect between species, which may be difficult to link to species diversity. However, such a negative effect can affect the shape of RADs through serrated frequency distributions by competitive quasi‐exclusion. In this case, nonpeak species can persist even under intense competitive pressures from peak species. One important factor is an existence of empty sites. If colonies never become extinct (i.e., *m* = 0), empty sites are absent in the habitat, in which the most superior species can persist exclusively. The continuous provision of empty sites allows species to avoid competitive exclusion that is caused by a limiting similarity between species with similar niches.

In our analysis, competition between similar species is a key factor that influences the pattern of SADs. Relationships between community structure and competition between ecologically similar species have been linked to phylogenies (Webb et al., 2002). Sugihara et al. (2003) conducted a meta‐analysis of empirical data on the interspecific similarities of various traits, compared to a broken stick model, which showed that symmetric branching in similarity dendrograms are negatively correlated with RAD evenness. Remarkably, we obtained a comparable relationship between similarity and evenness based on our colonization model, considering the shape of the trade‐off function as a determinant of the dendrogram. With saturated trade‐off functions, a concave trade‐off shape could result in an asymmetric branching pattern in a similarity dendrogram; species with low competitive ability are likely to coalesce due to their trait similarities that are relevant to fecundity, whereas species with high competitive ability tend to place at outside branches due to significantly different traits. Inversely, when the trade‐off function is nearly linear, the branches become more symmetric. Sugihara et al. measured evenness using the probability of an interspecific encounter (Hurlbert, 1971; Sugihara et al., 2003), which can be approximated to Simpson's diversity index by assuming an infinite number of individuals. In our analysis, Simpson's diversity index tends to decrease with a concave trade‐off function as 0.876, 0.783, and 0.544 in Figure 1d–f and 0.975, 0.962, and 0.945 in Figure 1g–i, respectively, where the difference decreases with increasing resolution. These tendencies suggest that the colonization process results in a trend consistent with the result of Sugihara et al., that is, a linearity of trade‐off function results in a symmetric similarity dendrogram and a relatively even RAD (Sugihara et al., 2003). In this case, the symmetry of the dendrogram might indirectly correlate with the RAD evenness, rather than the former causing the latter.

Previous theories on species and individual replacement processes showed that species diversity can be maintained through a continuous dynamic transition of species composition, for example, the lottery model and neutral theory (Chesson & Warner, 1981; Hubbell, 2001). However, our analyses indicate that the replacement of an individual or colony can lead to a state of community equilibrium and multiple species coexisting with trade‐offs between species abilities. We examined the model predictions by referring to studies on animal communities, although they may be applicable to other communities, such as tree species diversity in forest ecosystems. Mature trees tend to be relatively tolerant against competition, whereas their seedlings compete for gaps created by disturbances (Brokaw & Busing, 2000). Some modification of the assumptions is necessary to fit the model to those conditions. Here, empty sites are re‐denoted as sites that cannot be colonized due to poor site condition or presence of nontree species. Disturbances can transform both empty and occupied sites into gaps at a rate of *g*. In a gap originating from an occupied site, seedlings of the former occupant would dominate in the floor, although it could be invaded by a propagule of the competitively superior species via pairwise competition. In the absence of an invasion by propagules, the gaps recover to their original conditions via seedling growth of the original species or the degradation of site condition due to the absence of seedlings. If the competitive juvenile period is brief, Eq. (1) can be modified as

(5) $$\frac{dp_{i}}{\mathrm{dt}}=qf_{i}p_{i}g\left(1‐\sum_{j=1}^{i}p_{j}\right)‐q\left(\sum_{j=1}^{i‐1}f_{j}p_{j}\right)gp_{i}‐mp_{i}.$$

In this equation, *m* represents the rate that occupied sites gradually decay to uninvadable empty sites. Since the structure of Eq. (5) is equivalent to Eq. (1), if *qg* is replaced by *q*′, the tree communities could display trends similar to the presented analysis. Besides the gap formations, the uninvadable empty sites are essential for this system (i.e., *m* > 0), without which the most competitively superior species would exclusively dominate. Importantly, Rees et al. reviewed papers suggesting competition–colonization trade‐offs in tree species and also showed a trade‐off between high‐light growth and low‐light survivorship in young individuals of temperate tree species (Rees et al., 2001). Therefore, our predictions could be applicable to the coexistence of tree species.

In this study, we focused on saturating functions of fecundity–colonization trade‐off. We considered that the saturating shape of trade‐offs is reasonable with maximum fecundity that is environmentally determined, although we examined other types of trade‐off functions. Under a constant *m*/*q* value, if the trade‐off function is convex, species with low competitive ability may not decline due to the advantage of relatively high fecundity, and the range of competitive ability for these species (i.e., *m*/*q* < *f*(*x*)) becomes narrow. According to those factors, species frequencies are unlikely to significantly decrease even at low competitive abilities, resulting in a less steep RAD curve, in which the second RAD phase could also be suppressed. However, under a sigmoidal trade‐off function with saturation, the effects of the convex portion become noninfluential, as the trends are similar to the concave trade‐off functions.

In this paper, we analyzed the role of competition–fecundity trade‐offs, although other types of trade‐offs may also affect species diversity, such as competition–mortality trade‐offs. The present study is a starting point to investigate the relationships between trade‐offs in species interactions and the structure of ecological communities.

## CONFLICTS OF INTEREST

We know of no conflicts of interest associated with this publication, and there has been no significant financial support for this work that could have influenced its outcome.

## AUTHOR CONTRIBUTIONS

Atsushi Yamauchi involved in conceptualization, methodology, formal analysis, investigation, writing—original draft and funding acquisition; Koichi Ito involved in methodology, validation, and writing—review and editing; Shota Shibasaki involved in methodology, validation, and writing—review and editing.

## ETHICS STATEMENT

We declare that this manuscript is original, has not been published before, and is not currently being considered for publication elsewhere.

## Supporting information

Fig S1‐3

Supplementary Material

Supplementary Material

## Data Availability

Mathematica notebook files: Zenodo https://doi.org/10.5281/zenodo.4558463
