# Supplementary material for: Colonization process determines species diversity via competitive quasi‐exclusion
Source: Ecol Evol. 2021 Mar 16;11(9):4470–80. doi: 10.1002/ece3.7342 (PMC8093681; doi:10.1002/ece3.7342)

CAPTIONS OF SUPPLEMENTARY FIGURES

Supplementary Figure 1. Examples of the effects of overall shifts of species competitiveness with fixed competitiveness intervals on species composition with identical conditions to Figure 1. Species positions are calculated by [original position] + [(interval of species competitiveness)/2] in the analysis of discrete competitiveness. This position shift is equivalent to a shift in trade-off function toward −(interval of species competitiveness)/2.

Supplementary Figure 2. Examples of the effects of small randomness in species competitiveness on species composition with identical conditions to Figure 1. Each specific competitive ability is chosen randomly within [original position] ± [(original interval of species competitiveness)/2] in the analysis of discrete competitiveness.

Supplementary Figure 3. Examples of the effects of large randomness in species competitiveness on species composition with identical conditions to Figure 1. Each specific competitive ability is chosen randomly within $0 \leq x \leq \hat{x} = 2.5$ in the analysis of discrete competitiveness.

Supplementary Figure 1

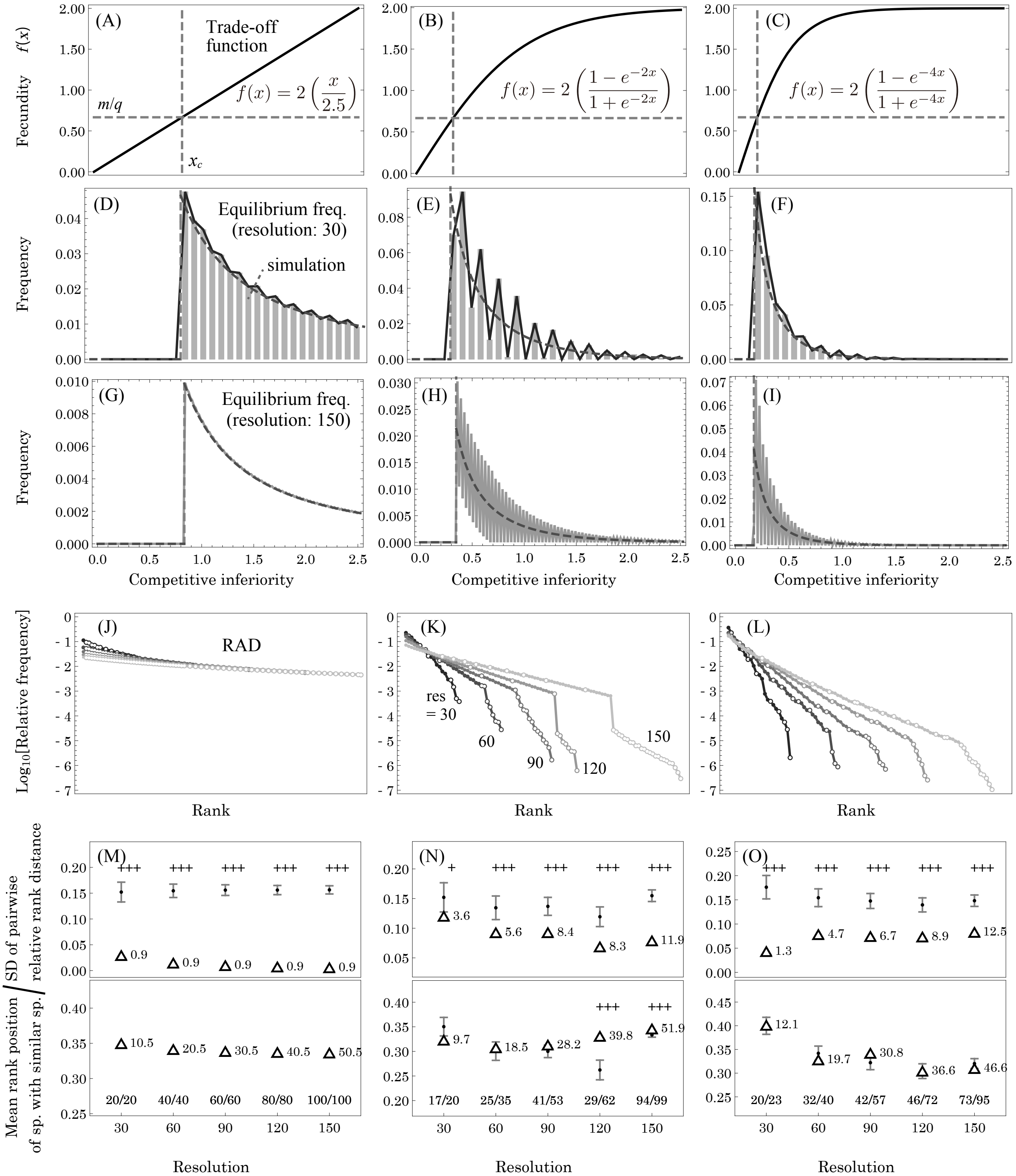

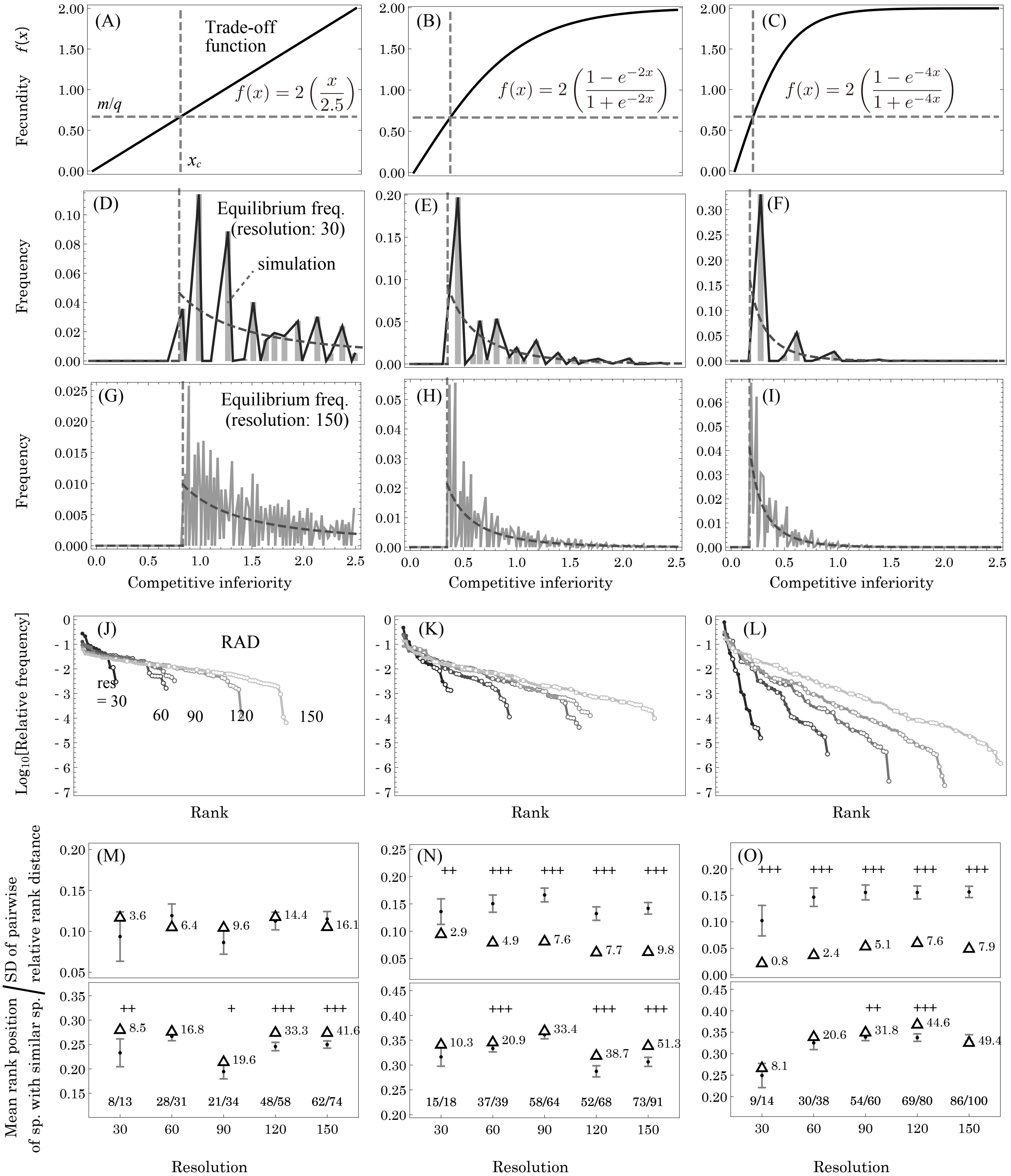
(A)
Trade-off function
$f(x) = 2\left(\frac{x}{2.5}\right)$
m/q
x_c
Fecundity $f(x)$
(B)
$f(x) = 2\left(\frac{1-e^{-2x}}{1+e^{-2x}}\right)$
(C)
$f(x) = 2\left(\frac{1-e^{-4x}}{1+e^{-4x}}\right)$
(D)
Equilibrium freq. (resolution: 30)
simulation
Frequency
(E)
(F)
(G)
Equilibrium freq. (resolution: 150)
(H)
(I)
Competitive inferiority
(J)
RAD
res = 30
60
90
120
150
Log_{10}[Relative frequency]
Rank
(K)
(L)
(M)
(N)
(O)
SD of pairwise relative rank distance
Mean rank position of sp. with similar sp.
8/13
28/31
21/34
48/58
62/74
15/18
37/39
58/64
52/68
73/91
9/14
30/38
54/60
69/80
86/100
Resolution

Supplementary Figure 3

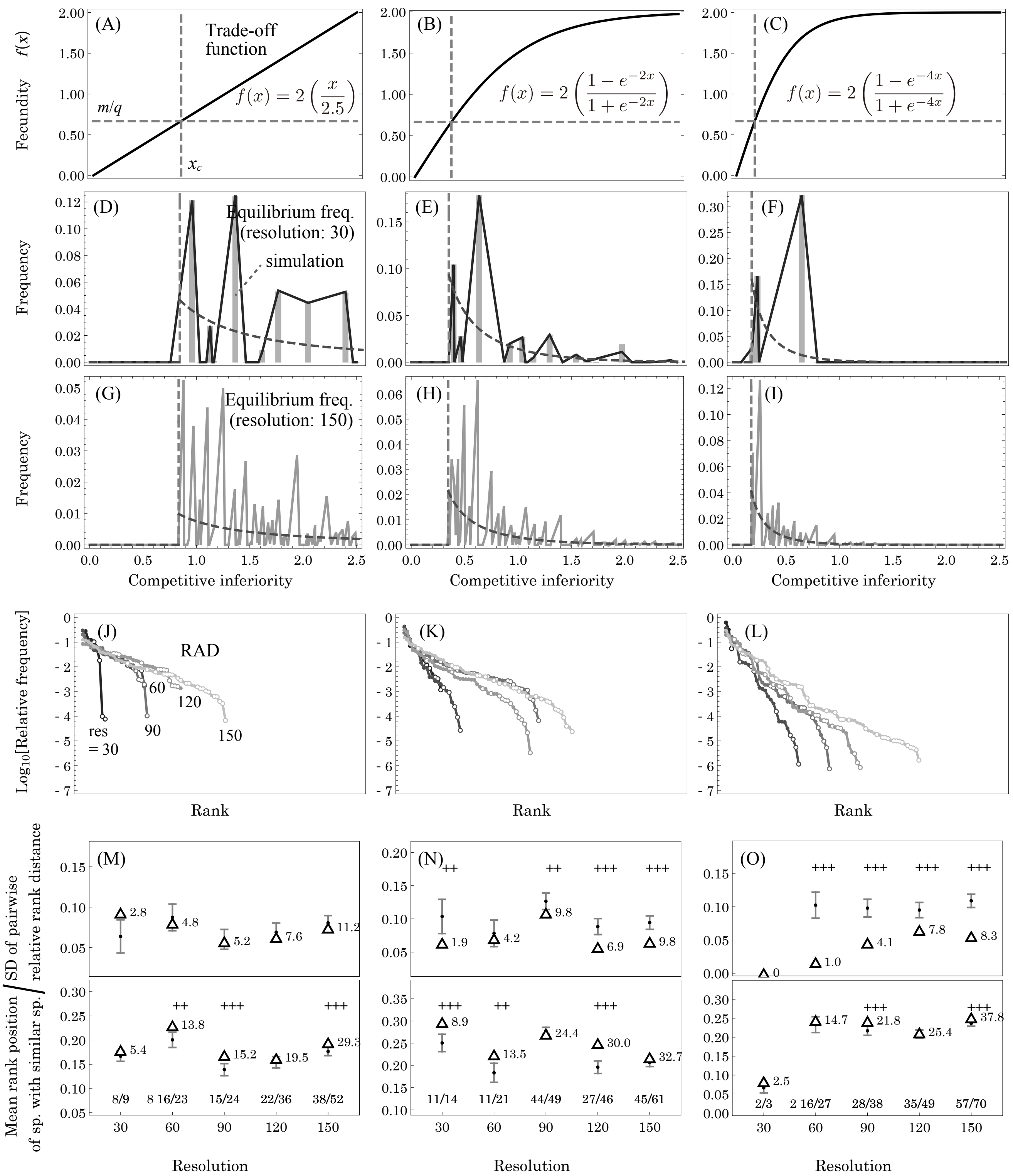

Supplement: Supplementary file 1 — Fig S1‐3 [file ECE3-11-4470-s001.pdf]
