# Supplementary material for: Colonization process determines species diversity via competitive quasi‐exclusion
Source: Ecol Evol. 2021 Mar 16;11(9):4470–80. doi: 10.1002/ece3.7342 (PMC8093681; doi:10.1002/ece3.7342)
Supplement: Supplementary file 3 — Supplementary Material [file ECE3-11-4470-s002.docx]

**APPENDIX A**

Trait distribution in competitiveness

From Eq. 1, a condition for the equilibrium state of the site frequency of *i*-th species (i.e., *dp_i_*/*dt* = 0) is:

$\frac{dp_{i}}{dt}=qf_{i}\left( 1-\sum_{j=1}^{i} p_{j} \right)-q\sum_{j=i}^{i-1} f_{j}p_{j}-m=0$ for ∀*i* = 1, 2, ..., *n*, (A1)

which can be rewritten as:

$p_{i}=1-\sum_{j=1}^{i-1} p_{j}-\frac{1}{f_{i}}\left( \sum_{j=1}^{i-1} f_{j}p_{j}+\frac{m}{q} \right)$ . (A2)

Therefore, the equilibrium frequency distribution can be:

$p_{i}=\left\{ \begin{matrix} 1-\sum_{j=1}^{i-1} p_{j}-\frac{1}{f_{i}}\left( \sum_{j=1}^{i-1} f_{j}p_{j}+\frac{m}{q} \right) & if it is positive \\ 0 & \mathrm{otherwise} \end{matrix} \right.$ . (A3)

This implies that a frequency of sites with *i*-th species can be determined from information of species with smaller numbering (i.e., *p_j_* for *j* < *i*). Namely, we can determine all *p_i_* by a forward recursive procedure from *i* = 1 to *n*. In this process, frequency may become positive first at *i* = $\hat{i}$. Since *p_j_* = 0 for *j* < $\hat{i}$ in this case, a species frequency at *i* = $\hat{i}$ can be:

$p_{\hat{i}}=1-\frac{1}{f_{\hat{i}}}\frac{m}{q}>0$ , (A4)

from Eq. A3; therefore,

$f_{\hat{i}}>\frac{m}{q}$ (A5)

must be satisfied.

**APPENDIX B**

Trait distribution under an approximation to continuous competitiveness

By approximately translating discrete competitiveness *i* to continuous value *x*, Eq. A1 is:

$\frac{dp}{dt}=qf(x)\left( 1-\int_{0}^{x} p(y)dy \right)-q\int_{0}^{x} f(y)p(y)dy-m=0$ . (B1)

By differentiating Eq. B1 with respect to *x*, we obtain:

$p\left( x \right)+\frac{1}{2}\frac{f^{'}\left( x \right)}{f\left( x \right)}\int_{x_{n}}^{x} p\left( y \right)dy-\frac{1}{2}\frac{f'(x)}{f(x)}=0$ . (B2)

By replacing an integration of *p*(*y*) by *P*(*x*), it can be rewritten as:

$P'(x)+\frac{1}{2}\frac{f'(x)}{f(x)}P(x)=\frac{1}{2}\frac{f'(x)}{f(x)}$ . (B3)

Since this is an ordinary first order differential equation for *P*(*x*), we can solve it as:

$P(x)=\left( \int\frac{1}{2}\frac{f'(x)}{f(x)}e^{\int\frac{1}{2}\frac{f'(x)}{f(x)}dx}dx+C \right)e^{-\int\frac{1}{2}\frac{f'(x)}{f(x)}dx}$

$=1+\frac{C}{\sqrt{f(x)}}$ , (B4)

where *C* is an integral constant. Thus,

$\int_{0}^{x} p(y)dy=1+\frac{C}{\sqrt{f(x)}}$ . (B5)

Suppose that strain frequency *p*(*x*) can be positive at *x_min_* ≤ *x*. In this case, substituting *x* = *x_min_* into Eq. B5, we obtain:

$\int_{0}^{x_{min}} p(y)dy=1+\frac{C}{\sqrt{f(x_{min})}}=0$ ; (B6a)

thus,

$C=-\sqrt{f(x_{min})}$ . (B6b)

Conversely, differentiating Eq. (B5) with respect *x*, we obtain:

$p\left( x \right)=-\frac{1}{2}\frac{f'(x)}{{f(x)}^{3/2}}C=\frac{1}{2}\frac{f'(x)}{{f(x)}^{3/2}}\sqrt{f(x_{min})}$ , (B7)

which can be positive only with *f*'(*x*) > 0, implying that a species can coexist within the range of a positive slope of fecundity, *f*(*x*). Note that there could be several regions with positive frequencies, each of which are bounded by specific *x_min_* and *x_max_*.

We can derive a minimum competitive inferiority, *x_c_*, below which any species cannot exist, i.e., *p*(*x*) = 0 for *x* < *x_c_*, and *p*(*x*) > 0 for *x* = *x_c_*. Substituting *x_c_* into *x*, Eq. B1 is *qf*(*x_c_*) − *m* = 0 due to two integral terms being 0; therefore, *f*(*x_c_*) = *m*/*q*. (Note that *f*(*x*) < *m*/*q* for *x* < *x_c_* because *dp*/*dt* < 0). In a case that *x_min_* coincides with *x_c_*, Eq. (B6b) can be rewritten as:

$C=-\sqrt{\frac{m}{q}}$ . (B8)

Therefore, a region that is lowermost bounded by *x_min_* = *x_c_* represents a frequency distribution,

$p\left( x \right)=\frac{1}{2}\frac{f'(x)}{{f(x)}^{3/2}}\sqrt{\frac{m}{q}}$ . (B9)

This region can be uppermost bounded by *x_max_*, above which *f*(*x*) is indefinite or *f*'(*x*) > 0 is violated.
